# Peripapillary and macular microvasculature in neovascular age-related macular degeneration in long-term and recently started anti-VEGF therapy versus healthy controls

**DOI:** 10.3389/fmed.2022.1080052

**Published:** 2023-01-09

**Authors:** Cengiz Türksever, Laura Hoffmann, Katja Hatz

**Affiliations:** ^1^Medical Retina Department, Vista Augenklinik Binningen, Binningen, Switzerland; ^2^Faculty of Medicine, University of Basel, Basel, Switzerland; ^3^Augenklinik, Charité – Universitätsmedizin Berlin, Berlin, Germany

**Keywords:** optical coherence tomography angiography, age-related macular degeneration, microvasculature, intraocular pressure, perfusion density, flux index

## Abstract

**Aim:**

To investigate the peripapillary and macular microvasculature in neovascular age-related macular degeneration (nAMD) in recently started versus long-term anti-vascular endothelial growth factor (VEGF) therapy and healthy controls.

**Methods:**

Eyes with nAMD treated in a treat-and-extend regimen were assigned to group 1 (<5 injections) or 2 (≥20 injections) whereas group 3 constituted the healthy age-matched controls. Blood flow signals were acquired using PLEX^®^ Elite 9000 swept-source optical coherence tomography angiography (OCTA) of the macular and peripapillary regions. Mean ganglion cell complex (GCC) thickness values were quantified using spectral-domain optical coherence tomography (SD-OCT).

**Results:**

Including 80 eyes whereof 40 controls, macular superficial perfusion density was significantly reduced in group 1 and 2 compared to controls (*p* < 0.001; *p* = 0.010) without a difference between groups 1 and 2. Peripapillary perfusion parameters did not correlate with post-operative intraocular pressure (IOP) or number of anti-VEGF injections. Mean peripapillary flux index was significantly lower in group 2 than in controls (*p* = 0.023) and significantly decreased in the nasal quadrants for both AMD groups compared to group 3 (*p* = 0.013; *p* < 0.001). Mean peripapillary perfusion density was significantly reduced in both AMD groups compared to controls (0.515 ± 0.02 versus 0.556 ± 0.03, *p* < 0.0001).

**Conclusion:**

Frequency of anti-VEGF treatment in nAMD and post-operative IOP showed no correlation with peripapillary perfusion parameters, but anti-VEGF treated nAMD patients exhibited partly altered peripapillary perfusion compared to healthy controls. Reduced macular perfusion density of the inner retina in anti-VEGF treated nAMD compared to healthy controls might be discussed as an anti-VEGF treatment effect or a characteristic of nAMD.

## Introduction

Neovascular age-related macular degeneration (nAMD) is the leading cause of blindness in elderly patients in industrialized countries (1). Currently, standard treatment of nAMD follows vascular endothelial growth factor (VEGF) inhibition using approved drugs such as ranibizumab, aflibercept, and brolucizumab in different treatment regimens. Efficacy and safety of the molecules were proved by large scale studies (2–5).

Short-term rise of intraocular pressure (IOP) is a well-recognized and very common complication of intravitreal anti-VEGF injection. A recent meta-analysis reported a significantly increased IOP on the day of injection with a normalization of IOP after 1 week (6). Some studies also suggest a sustained rise in IOP after 2 years of treatment in repeatedly injected eyes (7, 8). Recurrent anti-VEGF injections have been associated with a significant reduction in retinal nerve fiber layer (RNFL) thickness after 12 months, yet its clinical relevance remains uncertain (6).

With increased IOP after intravitreal anti-VEGF injection possible consequences on retinal perfusion have been assessed by retinal oximetry. An altered retinal oxygen metabolism has been discovered in different retinal diseases (9). A reduced oxygen extraction, which corresponds to the arteriovenous difference (AVD) in oxygen saturation, compared to healthy controls has been shown in nAMD whereas the vessel diameters were similar (10). While the impact of the choroidal vasculature in nAMD has been well-established, a decrease in blood flow velocity in retinal arteries as measured by the retinal function imager has been reported in patients with nAMD as well (11).

The development of optical coherence tomography angiography (OCTA) enables a non-invasive analysis of the vessel density in the different layers of the retina and the choriocapillaris (CC). A parapapillary microvascular dropout correlating with the reduced RNFL thickness has been described in glaucomatous eyes (12). A retrospective analysis comparing eyes with exudative AMD and non-exudative AMD revealed a significantly decreased retinal vessel density in the superficial capillary plexus in the group with exudative AMD, especially in the parafoveal region (13). However, in nAMD after a loading phase of anti-VEGF injections the macular vessel density of the retina and the CC remained unchanged compared to baseline (14).

Currently, there is still limited data on the effects of repeated intravitreal injections on peripapillary and macular retinal vessel density. Therefore, the aim of this study was to examine and compare OCTA blood flow indices of the macular and optic nerve head (ONH) regions in patients with nAMD with recently started and long-term anti-VEGF treatment, respectively, and healthy controls.

## Materials and methods

This cross-sectional, single-visit study was conducted at Vista Augenklinik Binningen, Switzerland. The study was approved by the local ethics committee (Ethikkommission Nordwestschweiz–EKNZ, EKNZ-No 2018-02043) and registered at clinicaltrials.gov (NCT 03833830). The research was conducted in accordance with the tenets of the Declaration of Helsinki and Good Clinical Practice (ICH-GCP).

All subjects gave written informed consent after information about the study. Patients with a clinical diagnosis of nAMD confirmed by a retina specialist and fulfilling the inclusion criteria were recruited consecutively at the Medical Retina department. Eligible patients were required to have been treated for sub- or juxtafoveal MNV due to nAMD with anti-VEGF intravitreal injections either for <5 times (group 1; short-term treatment eyes) or at least 20 times (group 2; long-term treatment eyes). Exclusion criteria were a diagnosis of glaucoma/ocular hypertension at baseline of anti-VEGF treatment, history of retinal vascular disorders like diabetic retinopathy, retinal vein/arterial occlusive disease, or uveitis, history of papillary disease which might interfere with interpretation of peripapillary imaging evaluation such as severe tilted disc, parapapillary MNV, papillary drusen, optic nerve neuritis, or papillary edema and inability to perform study imaging of sufficient quality. For each patient, only one eye was selected and included in the study. Age- and sex-matched healthy volunteers were recruited at the General Ophthalmology department. Study examinations took place between December 2018 and January 2021. Intravitreal injections (0.5 mg ranibizumab–Lucentis^®^, Novartis, Basel, Switzerland; 2 mg aflibercept – Eylea^®^, Bayer, Basel, Switzerland) were performed following a standardized procedure (15) in an operating room setting.

**FIGURE 1:**
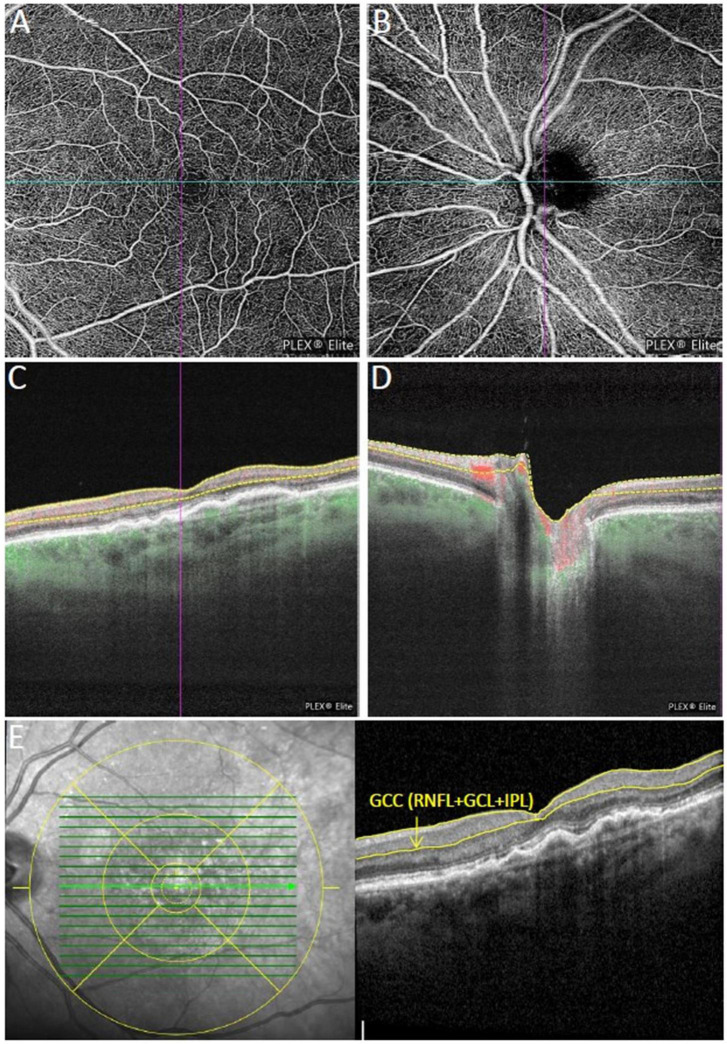
Multimodal imaging of an eye with neovascular age-related macular degeneration (nAMD) previously treated with four intravitreal injections of aflibercept. **(A,B)** Show the macular and peripapillary optical coherence tomography angiography (OCTA) 6 × 6 mm en-face scans graded as qualitatively very good. Vessel density measurement for superficial **(C)**, and deep retinal layer were performed separately in the macular region, while vessel density and flux index measurements of the optic nerve head (ONH) were evaluated within the 2 mm annulus of the 6 × 6 mm ONH-centered scan **(D)**. **(E)** Macular ganglion cell complex (GCC) thickness in the central 3 mm (retinal nerve fiber layer (RNFL) + ganglion cell layer + inner plexiform layer) were measured using the segmentation tool with an ETDRS grid overlay centered on the fovea.

For each patient and healthy subject one study visit was performed including best-corrected visual acuity measurement (BCVA), dilated biomicroscopic fundus examination, IOP measurement prior and 10 min after the intravitreal injection, spectral-domain optical coherence tomography (SD-OCT) (Spectralis, Heidelberg Engineering, Inc., Heidelberg, Germany) and swept source OCTA (SS-OCTA) (PLEX Elite 9000, Carl Zeiss Meditec, Dublin, USA) (Figure 1). Apart from BCVA measurement all examinations were performed in mydriatic pupil state. SD-OCT scans were acquired using an established protocol comprising 19 horizontal scans of 6 mm length (volume scan) in follow-up modus and a 6 mm star scan centered on the fovea. After manual correction of segmentation errors, macular SD-OCT scans were analyzed for central retinal thickness (CRT), characteristics of sub- and intraretinal fluid and vitreomacular interface. Circumpapillary RNFL thickness and macular ganglion cell complex (GCC) thickness (RNFL + ganglion cell layer + inner plexiform layer) were measured within the central 3 mm using an ETDRS grid overlay (Figure 1E). All SD-OCT images were reviewed and assessed by the same observer (CT) following standard evaluation protocols. IOP elevation following the intravitreal injection was calculated as the difference in IOP value prior and 10 min after the injection.

**FIGURE 2:**
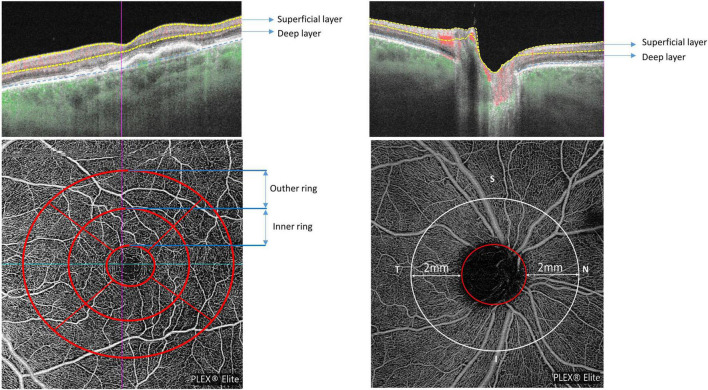
Macular and peripapillary optical coherence tomography angiography (OCTA) 6 × 6 mm scans with B-scans **(top row)** showing the location of superficial and deep layers and en-face scans **(bottom row)** with macular ETDRS grid **(left)** and the 2 mm annulus of the 6 × 6 mm optic nerve head (ONH)-centered scan **(right)**.

The OCTA scan protocols for imaging vessel density and peripapillary flux index included a 6 × 6 mm scan centered on the fovea and a 6 × 6 mm scan centered on the ONH (Figure 2). Each 6 × 6 mm section consisted of 500 A-scans per B-scan. Vessel density measurement for superficial and deep retinal layer were performed separately in the macular region. Vessel density and flux index measurements within the 2 mm annulus of the 6 × 6 mm ONH-centered scan were evaluated.

Quality assessment of each SS-OCTA measurement was performed by an OCTA-experienced ophthalmologist (CT) and similarly graded as previously described by Ali et al. (16):

• Grade 0 (degraded image quality): vascular structures are hardly visible, loss of signal, band of motion artefacts (excluded from study).
• Grade I (good image quality): visible vascular structures, no or very decent motion artefacts, visible vascular margins at the 6 mm central part of the OCTA image.
• Grade II (very good image quality): continuous vascular structures, very subtle lines of motion artefact, vessels are clearly visible.
• Grade III (excellent image quality): continuous vascular structures, no lines of motion artefact, vessels are perfectly visible.

In addition, blood pressure, body mass-index (BMI), and pulse pressure were measured and calculated for each subject.

### Statistical analysis

Statistical analyses were carried out with SPSS version 24.0 for Windows (SPSS, Chicago, IL, USA). Continuous variables were described as mean ± standard deviation (SD) or percentages. For comparison of means between different subgroups an ANOVA analysis applying a Bonferroni adjustment were performed. Correlation analyses (univariate) followed Pearson’s test. A *p*-value < 0.05 was considered statistically significant.

## Results

While a total of 94 eyes of 94 subjects were recruited in this study, 14 eyes were excluded due to insufficient (grade 0) image quality. In total, 80 eyes of 80 subjects with good, very good or excellent OCTA image quality (grade 1, 2, or 3) were included for further analysis. Forty eyes were included in the nAMD groups whereof 19 eyes in group 1 (<5 injections) and 21 eyes in group 2 (>20 anti-VEGF injections) at the time of the study visit as well as 40 eyes in the control group. Twenty-two eyes (55%) received ranibizumab, 6 (15%) aflibercept, and 12 (30%) mixed agents. Treatment distribution in group 1 was 94.7% (*n* = 18) ranibizumab and 5.3% (*n* = 1) aflibercept and in group 2 19% (*n* = 4) ranibizumab, 23.8% (*n* = 5) aflibercept, and 57.1% (*n* = 12) mixed agents. Demographic and functional characteristics are summarized in Table 1.

**TABLE 1:** Demographic and hemodynamic characteristics of subjects.

**Groups**	**Number of subjects**	**Mean age, years (±SD)**	**Female sex (*n*, %)**	**Mean BCVA (±SD),** **(ETDRS letters)**	**Mean IOP change after injection; mmHg**
**Demographic characteristics**					
Controls	40	78.4 ± 5.1	21 (52.5)	81.4 ± 5.4	
Group 1	19	79.0 ± 6.6	12 (63.1)	74.7 ± 13.6	7.57 ± 6.9
Group 2	21	77.9 ± 7.2	9 (42.9)	70.0 ± 20.6	10.0 ± 8.1
	**Mean ± SD**		**Comparison between groups**		***p*-values between groups** ***post-hoc* test with ANOVA**
**Hemodynamic characteristics**					
BMI (kg/mm^2^)	Controls	24.6 ± 3.35	Controls	Group 1	1.000
	Group 1	24.4 ± 4.06	Controls	Group 2	1.000
	Group 2	25.2 ± 3.26	Group 1	Group 2	1.000
Pulse pressure (mmHg)	Controls	61 ± 15.68	Controls	Group 1	0.863
	Group 1	55.9 ± 21.35	Controls	Group 2	**0.012**
	Group 2	74.6 ± 15.03	Group 1	Group 2	**0.003**
Systolic blood pressure (mmHg)	Controls	143 ± 16.66	Controls	Group 1	0.365
	Group 1	134.5 ± 26.58	Controls	Group 2	**0.048**
	Group 2	155.9 ± 16.89	Group 1	Group 2	0.003
Diastolic blood pressure (mmHg)	Controls	81.9 ± 8.36	Controls	Group 1	0.793
	Group 1	78.6 ± 11.23	Controls	Group 2	1.000
	Group 2	81.4 ± 13	Group 1	Group 2	1.000
Heart rate per minute	Controls	69.7 ± 7.96	Controls	Group 1	*0.092*
	Group 1	76 ± 12.41	Controls	Group 2	1.000
	Group 2	70.7 ± 12.15	Group 1	Group 2	0.329

Bold values represented the significant *p*-values.

The mean number of prior intravitreal injections in eyes with nAMD was 19.97 ± 20.56 (range 1– 85) with on average 2.79 ± 1.03 in group 1 and 35.52 ± 17.00 in group 2. The OCTA examination in groups 1 and 2 took place after on average 41.45 ± 16.5 days following the last intravitreal injection. A total of 13 (32.5%) out of 40 eyes showed an elevation of IOP > 25 mmHg 10 min after the intravitreal injection with a maximal increase of 25 mmHg following the injection. Interestingly, in one eye IOP was significantly reduced from 18 to 6 mmHg after the injection, probably due to a subtle leakage in a very thin sclera after 31 intravitreal injections.

### Hemodynamics in patients with nAMD

Hemodynamic parameters are shown in Table 1. While there was no significant difference between the subgroups in mean BMI, heart rate per minute, and diastolic blood pressure, mean systolic blood pressure was significantly higher in group 2 compared to group 1 and the controls (*p* = 0.048; *p* = 0.003, respectively). Likewise, pulse pressure was significantly higher in group 2 than in group 1 (*p* = 0.003) and controls (*p* = 0.012). The BMI of the subjects was equally distributed with 11 and 14 cases of overweight as well as 5 and 4 cases of obesity in the control group and the nAMD subgroups, respectively.

### Spectral domain optical coherence tomography of the optic disc and macula

Main OCT parameters are summarized in Table 2.

**TABLE 2:** Main optical coherence tomography (OCT) parameters.

SD-OCT parameters	Mean ± SD		Comparison between groups		*p*-values between groups *post-hoc* test with ANOVA
Central retinal thickness (μm)	Controls	278.1 ± 23.9	Controls	Group 1	0.603
	Group 1	294.1 ± 74.1	Controls	Group 2	1.000
	Group 2	270.1 ± 40.4	Group 1	Group 2	0.279
Mean peripapillary RNFL thickness (μm)	Controls	83.3 ± 7.5	Controls	Group 1	0.776
	Group 1	80.7 ± 9.1	Controls	Group 2	0.683
	Group 2	86.0 ± 8.6	Group 1	Group 2	0.136
Mean macular GCC thickness in central 3 mm (μm)	Controls	99.3 ± 7.8	Controls	Group 1	1
	Group 1	97.7 ± 9.9	Controls	Group 2	0.190
	Group 2	103.8 ± 9.5	Group 1	Group 2	0.093

In the nAMD groups, 20 eyes (50%) exhibited macular subretinal and 5 (12.5%) intraretinal fluid. The mean peripapillary RNFL was not significantly different between the subgroups (Table 2). There was no correlation between RNFL thickness and number of anti-VEGF injections in the subgroups (*r* = 0.215; *p* = 0.183). The mean GGC in the central 3 mm was thicker in group 2 compared to group 1 (103.8 μm ± 9.49 versus 97.68 μm ± 9.95; *p* = 0.093), particularly in the inferior part of the macula (120.95 μm ± 12.93 versus 110.42 μm ± 14.93, *p* = 0.018).

### OCTA image quality

All images of the 80 included patients fulfilled the eligibility criteria for inclusion to study. However, due to fixation difficulties the image quality was better in controls than in eyes with nAMD. In the latter macular OCTA image quality was graded as excellent in 13 eyes (32.5%), as very good in 18 eyes (45%), and as good in 9 eyes (22.5%). Regarding the peripapillary area OCTA image quality was excellent in 12 eyes (30%), very good in 16 eyes (40%), and good in 12 eyes (30%). In the control group 26 eyes (65%) had excellent macular OCTA image quality, 10 eyes had very good (25%), and only 4 eyes (10%) showed good image quality. In the peripapillary region, the distribution of excellent, very good, and good image quality was 60, 30, and 10%, respectively. While the proportion of pseudophakic eyes was comparable between the nAMD groups and the control group (55 versus 60%), no subject presented with significant cataract formation.

### Macular perfusion characteristics

Perfusion characteristics of the macula are shown in Table 3.

**TABLE 3:** Macular perfusion characteristics.

Macular perfusion density based on SS-OCTA	Mean ± SD		Comparison between groups		Mean difference	*p*-values between groups* *post-hoc* test with ANOVA
**Superficial perfusion density of the macula**						
Central 3 mm	Controls	0.409 ± 0.035	Controls	Group 1	0.052	**0.000**
	Group 1	0.357 ± 0.047	Controls	Group 2	0.032	**0.010**
	Group 2	0.376 ± 0.04	Group 1	Group 2	−0.02	0.374
Central 6 mm	Controls	0.421 ± 0.036	Controls	Group 1	0.043	**0.001**
	Group 1	0.378 ± 0.043	Controls	Group 2	0.039	**0.001**
	Group 2	0.382 ± 0.039	Group 1	Group 2	−0.003	1.000
**Deep perfusion density of the macula**						
Central 3 mm	Controls	0.234 ± 0.064	Controls	Group 1	0.019	1.000
	Group 1	0.215 ± 0.095	Controls	Group 2	0.009	1.000
	Group 2	0.225 ± 0.093	Group 1	Group 2	−0.01	1.000
Central 6 mm	Controls	0.196 ± 0.060	Controls	Group 1	−0.024	0.776
	Group 1	0.220 ± 0.095	Controls	Group 2	−0.01	1.000
	Group 2	0.210 ± 0.090	Group 1	Group 2	0.014	1.000

*p-values adjusted with the Bonferroni adjustment.

Bold values represented the significant *p*-values.

The deep perfusion density in both the central 3 and 6 mm were not significantly different between the three subgroups. However, the superficial perfusion density in the central 3 and 6 mm were found to be higher in controls than in group 1 (*p* < 0.0001; *p* = 0.001, respectively) and group 2 (*p* = 0.01; *p* = 0.001, respectively) (Table 3). Furthermore, in eyes with nAMD the presence of subretinal fluid within the 6 mm ETDRS grid (*N* = 20) was associated with a better superficial macular perfusion density in the central 6 mm (*p* = 0.036) compared to eyes without subretinal fluid (*n* = 20).

### Peripapillary perfusion characteristics

The mean average peripapillary flux index was significantly reduced in group 2 compared to the control group (0.377 ± 0.04 versus 0.415 ± 0.06; *p* = 0.023) (see Table 4).

**TABLE 4:** Peripapillary perfusion characteristics.

Peripapillary flux index based on SS-OCTA	Mean ± SD		Comparison between groups		*p*-values between groups* *post-hoc* test with ANOVA
Average	Controls	0.415 ± 0.056	Controls	Group 1	0.383
	Group 1	0.392 ± 0.049	Controls	Group 2	**0.023**
	Group 2	0.377 ± 0.042	Group 1	Group 2	0.995
Temporal	Controls	0.402 ± 0.052	Controls	Group 1	*0.099*
	Group 1	0.375 ± 0.034	Controls	Group 2	**0.012**
	Group 2	0.367 ± 0.036	Group 1	Group 2	1.000
Superior	Controls	0.425 ± 0.070	Controls	Group 1	1.000
	Group 1	0.414 ± 0.067	Controls	Group 2	0.309
	Group 2	0.395 ± 0.061	Group 1	Group 2	1.000
Nasal	Controls	0.407 ± 0.048	Controls	Group 1	**0.013**
	Group 1	0.371 ± 0.045	Controls	Group 2	**0.000**
	Group 2	0.354 ± 0.033	Group 1	Group 2	0.668
Inferior	Controls	0.434 ± 0.070	Controls	Group 1	0.602
	Group 1	0.410 ± 0.062	Controls	Group 2	*0.065*
	Group 2	0.392 ± 0.059	Group 1	Group 2	1.000

*p-values adjusted with the Bonferroni adjustment.

Bold values represented the significant *p*-values.

The biggest differences when comparing group 2 and the controls were observed for the nasal peripapillary flux index (0.354 ± 0.03 versus 0.407 ± 0.05; *p* < 0.0001) and the temporal flux index of the ONH (*p* = 0.012). While in group 1 mean average peripapillary flux index was not significantly lower compared to the controls (0.392 ± 0.05 versus 0.415 ± 0.06, *p* = 0.383), the nasal peripapillary flux index was also significantly decreased compared to controls (*p* = 0.013). Interestingly, there was no significant difference in the flux indices between the two nAMD subgroups (0.371 ± 0.05 versus 0.407 ± 0.05, *p* = 0.995). Furthermore, there was no correlation between the peripapillary flux index in eyes with nAMD and neither IOP elevation after the injection nor the number of anti-VEGF injections received (*r* = 0.130, *p* = 0.425; *r* = −0.135, *p* = 0.405). The time period after the last injection was not significantly associated with neither peripapillary nor macular microvascular parameters.

The mean average peripapillary perfusion density was significantly reduced in the AMD groups compared to controls (0.515 ± 0.02 versus 0.556 ± 0.03, *p* < 0.0001) while there was no difference between group 1 and 2 (*p* = 0.779). This difference in peripapillary perfusion density between AMD subjects and controls persisted in all four peripapillary subquadrants (*p* < 0.01).

## Discussion

While several studies evaluated macular microvasculature in macular diseases treated with intravitreal anti-VEGF injections (13, 14) to the best of our knowledge, this study is the first to evaluate peripapillary microvasculature in eyes with long-term versus recently started anti-VEGF therapy for nAMD compared to healthy controls.

Previous OCTA studies described the impact of OCTA image quality on the measurement itself (17, 18). Therefore, in our study we only included eyes with good, very good or excellent OCTA images based on the quality assessment as described by Ali et al. (16). In our study, better macular and peripapillary image quality was found in healthy controls compared to eyes with nAMD. This difference probably resulted from the impaired fixation ability due to the reduced visual acuity and/or central scotomas in nAMD patients.

While the subgroups were well balanced in terms of age, sex and BMI, an increased systolic blood pressure, and pulse pressure in nAMD patients following long-term anti-VEGF therapy compared to controls and nAMD patients with recently started intravitreal anti-VEGF treatment was noted. Higher pulse pressure is defined as an increased arterial stiffness and is a well-known risk factor for several cardiovascular diseases. While higher pulse pressure has been associated with an increased risk for late nAMD, (19) it cannot be excluded to be a systemic side-effect of the long-term anti-VEGF therapy (20). Furthermore, the FEAR study described a transient increase in blood pressure associated with intravitreal injections attributed to an anxiety response which may constitute a cardiovascular risk factor in frequent injections (21).

Due to the acute IOP changes caused by intravitreal injections, an acute decrease in angiographic PD in the overall optic nerve head, especially temporally, has been shown (22). A recent meta-analysis reported on a reduction of peripapillary RNFL thickness after recurrent injection of anti-VEGF with questionable clinical relevance (6). However, studies assessing the impact of repeated IVIs on the peripapillary microvasculature are lacking so far. In our study mean peripapillary perfusion density in all subquadrants was significantly reduced in both AMD groups compared to controls without a difference between the AMD eyes. Further, the average peripapillary flux index in eyes with frequent injections was significantly reduced, preferentially in the temporal and nasal area, compared to the controls in the absence of a correlation with neither IOP elevation after the injection nor the number of anti-VEGF injections received. No differences in peripapillary RNFL thickness between the subgroups were found. While several risk factors for a sustained and delayed elevation of IOP in eyes undergoing anti-VEGF injections like the cumulative number of IVIs, intervals between injections and preexisting glaucoma have been described, (23) the etiology of the decrease in peripapillary flux index in frequently treated eyes likely seems multifactorial. Hence, altered peripapillary perfusion may as well represent a characteristic of AMD itself.

In our study, superficial but not deep macular perfusion density were reduced in both nAMD groups compared to controls supporting previously reported results showing decreased superficial macular vessel density in nAMD compared to healthy controls and non-exudative AMD (13). Lee at al. showed a decreased macular vessel density with age but failed to report a linear correlation with the number of previous intravitreal injections (13). Likewise in our study, no difference in perfusion density was observed between long-term and short-term treated nAMD groups. Given the cross-sectional character of our study the pathogenesis of the reduced superficial perfusion density remains unclear but a possible explanations might include a vasoconstrictor effect of anti-VEGF therapy with immediate effect upon the first injection as well as a characteristic of AMD itself. Rosen et al. described a decrease of the superficial parafoveal perfusion density immediately after intravitreal injection (22). Hikichi et al. reported a deterioration of the vessel density of the deep capillary plexus and the CC but not the superficial capillary plexus with long-term anti-VEGF therapy (24). However, given the different OCTA algorithms applying variable boundaries for layer segmentation, comparisons between studies must consider the differing measurement methodologies (25).

It has been suggested that in nAMD proportionally less oxygen is extracted by the retinal vessels as a consequence of decreased oxygen demand by the atrophic tissue. However, minutes after anti-VEGF injection, a significant increase in AVD has been observed in patients with nAMD while vessel diameter remained unchanged (26). This short-term elevation in oxygen consumption is attributed either to an increased metabolism or a reduced blood flow due to a transient peak in IOP. However, in healthy controls a similar rise in AVD after an induced increase in IOP has been observed while retinal blood flow remained stable (27). Mendrinos et al. reported on a long-term a reduction in retinal artery diameter after single or repeated intravitreal anti-VEGF injections in nAMD (28). These findings could suggest a pharmacological vasoconstrictor effect of anti-VEGF treatment.

Another novel finding of our study consists in the better macular perfusion density in eyes with central subretinal fluid. Possibly, these eyes exhibit greater nAMD activity resulting in an increased oxygen demand. Beforehand, subretinal fluid has been discussed to be associated with an improved long-term BCVA outcome (29). Likewise, the FLUID study suggested to tolerate SRF while shifting toward an intraretinal fluid focused disease management (30).

Limitations of this study are its cross-sectional character rather than a long-term follow-up study and the limited number of patients. On the other hand, strengths of our study consisted in the recruiting of a sex- and age-matched healthy control group and the exclusion of images with poor image quality.

To conclude, we report an increased pulse pressure in long-term anti-VEGF treated subjects and decreased macular superficial perfusion density in nAMD eyes which might be linked to systemic and local side-effects of the treatment or reflect characteristics of nAMD. While alterations in the peripapillary microvasculature were observed partly in all nAMD, but especially in frequently treated eyes, neither a correlation to post-operative IOP elevation nor number of previous injections could be established. Due to the cross-sectional character of the study definite conclusion concerning the pathogenesis of the observed alterations cannot be deduced. However, closer clinical evaluation of patients in need of frequent intravitreal injections over the long-term is advisable. Therefore, larger prospective studies including possible confounders are warranted to assess the impact of repeated intravitreal injections on the retinal microvasculature and possible cardiovascular side-effects.

## Data availability statement

On-request anonymized data can be shared if local ethics committee agrees. Requests to access the datasets should be directed to KH, katja.hatz@vista.ch.

## Ethics statement

The studies involving human participants were reviewed and approved by Ethikkommission Nordwestschweiz EKNZ. The patients/participants provided their written informed consent to participate in this study.

## Author contributions

CT: study concept, acquisition and interpretation of data, statistics, and writing of manuscript. LH: acquisition and interpretation of data, statistics, and writing of the manuscript. KH: study concept, supervision, and revision of the manuscript. All authors critically reviewed the manuscript and approved the final version.
